# 2-(6-Bromo-3-pyrid­yl)-8-methyl­imidazo[1,2-*a*]pyrazine

**DOI:** 10.1107/S1600536810022993

**Published:** 2010-06-23

**Authors:** Buwen Huang, Eugene Rui, Martin Wythes, Pei-Pei Kung, Curtis Moore, Arnold L. Rheingold, Alex Yanovsky

**Affiliations:** a Pfizer Global Research and Development, La Jolla Labs, 10770 Science Center Drive, San Diego, CA 92121, USA; b Department of Chemistry and Biochemistry, University of California, San Diego, 9500 Gilman Drive, La Jolla, CA 92093, USA

## Abstract

The structure of the title compound, C_12_H_9_BrN_4_, prepared by the reaction of 2-bromo-1-(6-bromo-3-pyrid­yl)ethanone with 2-amino-3-methyl­pyrazine indicates that the compound with the bromo­pyridyl substituent at position 2 of the imidazopyrazine fused-ring system represents the major product of this reaction. The plane of the pyridine ring forms a dihedral angle of 16.2 (2)° with the essentially planar (r.m.s. deviation = 0.006 Å) imidazopyrazine system. In the crystal, mol­ecules are linked by weak C—H⋯N inter­actions.

## Related literature

For the structure of the related imidazo(1,2-*a*)pyrazine deivative, see: Lumma & Springer (1981 ▶).
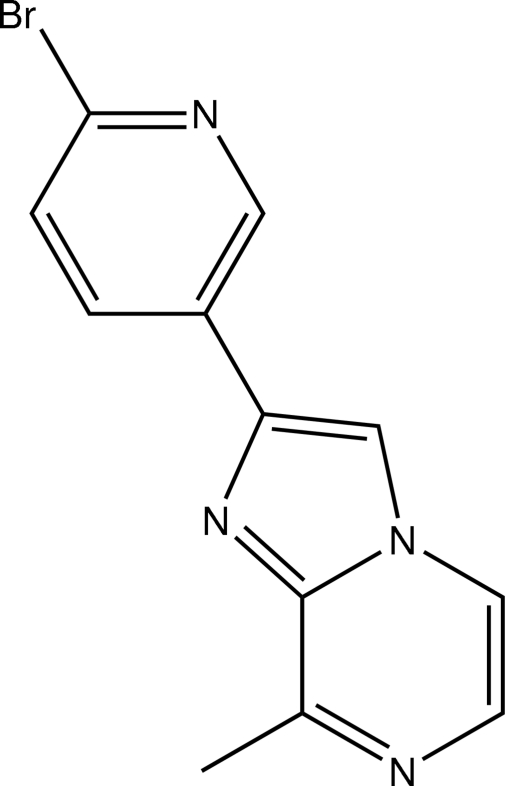


## Experimental

### 

#### Crystal data

C_12_H_9_BrN_4_
*M* *_r_* = 289.14
Monoclinic, 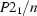

*a* = 3.9007 (14) Å
*b* = 13.545 (5) Å
*c* = 20.673 (8) Å
β = 93.059 (5)°
*V* = 1090.7 (7) Å^3^
*Z* = 4
Mo *K*α radiation
μ = 3.75 mm^−1^
*T* = 100 K
0.27 × 0.11 × 0.05 mm

#### Data collection

Bruker APEXII CCD diffractometer
Absorption correction: multi-scan (*SADABS*; Bruker, 2001 ▶) *T*_min_ = 0.431, *T*_max_ = 0.835
19939 measured reflections
2668 independent reflections
1887 reflections with *I* > 2σ(*I*)
*R*_int_ = 0.085

#### Refinement

*R*[*F* ^2^ > 2σ(*F* ^2^)] = 0.057
*wR*(*F* ^2^) = 0.154
*S* = 1.05
2668 reflections
155 parameters
H-atom parameters constrained
Δρ_max_ = 1.23 e Å^−3^
Δρ_min_ = −1.30 e Å^−3^

### 

Data collection: *APEX2* (Bruker, 2007 ▶); cell refinement: *SAINT* (Bruker, 2007 ▶); data reduction: *SAINT*; program(s) used to solve structure: *SHELXS97* (Sheldrick, 2008 ▶); program(s) used to refine structure: *SHELXL97* (Sheldrick, 2008 ▶); molecular graphics: *SHELXTL* (Sheldrick, 2008 ▶); software used to prepare material for publication: *SHELXTL*.

## Supplementary Material

Crystal structure: contains datablocks global, I. DOI: 10.1107/S1600536810022993/hb5478sup1.cif

Structure factors: contains datablocks I. DOI: 10.1107/S1600536810022993/hb5478Isup2.hkl

Additional supplementary materials:  crystallographic information; 3D view; checkCIF report

## Figures and Tables

**Table 1:** Hydrogen-bond geometry (Å, °)

*D*—H⋯*A*	*D*—H	H⋯*A*	*D*⋯*A*	*D*—H⋯*A*
C7—H7⋯N1^i^	0.95	2.52	3.438 (6)	163
C10—H10⋯N2^ii^	0.95	2.60	3.484 (7)	156

Symmetry codes: (i) 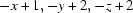; (ii) 
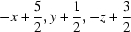
.

**Fig. 1.**
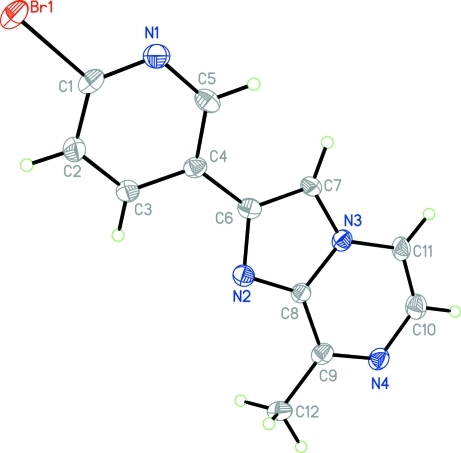
Molecular structure of the title compound, showing 50% probability displacement ellipsoids. H atoms are drawn as circles of arbitrary small radius.

## supplementary crystallographic information

### Comment

The reaction of 2-bromo-1-(6-bromo3-pyridyl)ethanone with 2-amino-3-methylpyrazine may potentially produce either 2- or 3-(6-bromo3-pyridyl)-8-methylimidazo[1,2-*a*]pyrazine. The present study shows that the compound with bromopyridyl substituent in position 2 of imidazopyrazine represents the major product of this reaction (Fig. 1).

The plane of the pyridine ring N1, C1—C5 forms the dihedral angle of 16.2 (2)° with the essentially planar imidazopyrazine system N2, N3, N4, C6—C11. Strange though it may seem, only one purely organic structure with non-protontated non-fused imidazo(1,2 - a)pyrazine system with only carbon substituents has been published heretofore (Lumma & Springer, 1981). The geometry of the bicyclic fragment in this structure is in good agreement with that of the title compound.

### Experimental

A mixture of 2-bromo-1-(6-bromo-3-pyridyl)-ethanone (2.70 g, 9.68 mmol), 2-amino-3-methylpyrazine (1.06 g, 9.68 mmol), and sodium bicarbonate (1.22 g, 14.5 mmol) in 40 ml of 2-propanol was heated at 80°C overnight. After cooling down to rt, the reaction mixture was concentrated to dryness. The resulting residue was partitioned between ethyl acetate (100 ml) and water (100 ml). The organic phase was washed with brine (1 × 100 ml), dried over sodium sulfate, concentrated to dryness, and purified by column chromatography with 0 --> 5% MeOH/EA to afford the desired product as a solid (1.25 g, 44.7% yield).

Colourless needles of (I) were grown by slow evaporation of an ethanol/dichloroethane solution.

### Refinement

All H atoms were placed in geometrically calculated positions (C—H 0.95 Å for aromatic and 0.98 Å for methyl H atoms, respectively) and included in the refinement in riding motion approximation. The *U*_iso_(H) were set to 1.2*U*_eq_ of the carrying atom (1.5*U*_eq_ for methyl H atoms). The maximum residual density peak 1.23 e/Å^3^ is located at a distance of 0.99 Å from the Br1 atom; the deepest hole -1.30 e/Å^3^ is at a distance of 0.78 Å from the Br1 atom.

### Crystal data

**Table:**

C_12_H_9_BrN_4_	*F*(000) = 576
*M**_r_* = 289.14	*D*_x_ = 1.761 Mg m^−^^3^
Monoclinic, *P*2_1_/*n*	Mo *K*α radiation, λ = 0.71073 Å
Hall symbol: -P 2yn	Cell parameters from 6098 reflections
*a* = 3.9007 (14) Å	θ = 3.3–27.2°
*b* = 13.545 (5) Å	µ = 3.75 mm^−^^1^
*c* = 20.673 (8) Å	*T* = 100 K
β = 93.059 (5)°	Needle, colorless
*V* = 1090.7 (7) Å^3^	0.27 × 0.11 × 0.05 mm
*Z* = 4	

### Data collection

**Table:**

Bruker APEXII CCD diffractometer	2668 independent reflections
Radiation source: fine-focus sealed tube	1887 reflections with *I* > 2σ(*I*)
graphite	*R*_int_ = 0.085
φ and ω scans	θ_max_ = 28.7°, θ_min_ = 1.8°
Absorption correction: multi-scan (*SADABS*; Bruker, 2001)	*h* = −5→5
*T*_min_ = 0.431, *T*_max_ = 0.835	*k* = −17→17
19939 measured reflections	*l* = −27→26

### Refinement

**Table:**

Refinement on *F*^2^	Primary atom site location: structure-invariant direct methods
Least-squares matrix: full	Secondary atom site location: difference Fourier map
*R*[*F*^2^ > 2σ(*F*^2^)] = 0.057	Hydrogen site location: inferred from neighbouring sites
*wR*(*F*^2^) = 0.154	H-atom parameters constrained
*S* = 1.05	*w* = 1/[σ^2^(*F*_o_^2^) + (0.0701*P*)^2^ + 3.9758*P*] where *P* = (*F*_o_^2^ + 2*F*_c_^2^)/3
2668 reflections	(Δ/σ)_max_ = 0.001
155 parameters	Δρ_max_ = 1.23 e Å^−^^3^
0 restraints	Δρ_min_ = −1.30 e Å^−^^3^

### Special details

**Table:**

Geometry. All e.s.d.'s (except the e.s.d. in the dihedral angle between two l.s. planes) are estimated using the full covariance matrix. The cell e.s.d.'s are taken into account individually in the estimation of e.s.d.'s in distances, angles and torsion angles; correlations between e.s.d.'s in cell parameters are only used when they are defined by crystal symmetry. An approximate (isotropic) treatment of cell e.s.d.'s is used for estimating e.s.d.'s involving l.s. planes.
Refinement. Refinement of *F*^2^ against ALL reflections. The weighted *R*-factor *wR* and goodness of fit *S* are based on *F*^2^, conventional *R*-factors *R* are based on *F*, with *F* set to zero for negative *F*^2^. The threshold expression of *F*^2^ > σ(*F*^2^) is used only for calculating *R*-factors(gt) *etc*. and is not relevant to the choice of reflections for refinement. *R*-factors based on *F*^2^ are statistically about twice as large as those based on *F*, and *R*- factors based on ALL data will be even larger.

### Fractional atomic coordinates and isotropic or equivalent isotropic displacement parameters (Å^2^)

**Table:**

	*x*	*y*	*z*	*U*_iso_*/*U*_eq_	
Br1	0.27478 (13)	0.63452 (4)	1.03763 (3)	0.0279 (2)	
N1	0.5377 (11)	0.8165 (3)	1.0087 (2)	0.0247 (9)	
N2	1.0290 (10)	0.9281 (3)	0.8097 (2)	0.0195 (8)	
N3	1.0640 (10)	1.0898 (3)	0.83304 (19)	0.0193 (8)	
N4	1.4045 (11)	1.1347 (3)	0.7232 (2)	0.0221 (9)	
C1	0.4653 (12)	0.7301 (4)	0.9823 (2)	0.0215 (10)	
C2	0.5223 (13)	0.7047 (4)	0.9189 (2)	0.0241 (11)	
H2	0.4687	0.6407	0.9026	0.029*	
C3	0.6590 (12)	0.7755 (4)	0.8807 (2)	0.0214 (10)	
H3	0.7011	0.7611	0.8369	0.026*	
C4	0.7370 (12)	0.8692 (3)	0.9062 (2)	0.0190 (10)	
C5	0.6736 (12)	0.8850 (4)	0.9704 (2)	0.0220 (10)	
H5	0.7290	0.9478	0.9887	0.026*	
C6	0.8818 (12)	0.9470 (3)	0.8672 (2)	0.0185 (10)	
C7	0.8997 (12)	1.0472 (3)	0.8822 (2)	0.0207 (10)	
H7	0.8150	1.0790	0.9191	0.025*	
C8	1.1395 (12)	1.0161 (3)	0.7898 (2)	0.0174 (9)	
C9	1.3167 (12)	1.0417 (4)	0.7337 (2)	0.0207 (10)	
C10	1.3238 (13)	1.2048 (4)	0.7677 (3)	0.0256 (11)	
H10	1.3897	1.2710	0.7598	0.031*	
C11	1.1591 (13)	1.1865 (3)	0.8214 (3)	0.0224 (10)	
H11	1.1088	1.2380	0.8507	0.027*	
C12	1.4005 (13)	0.9642 (4)	0.6864 (2)	0.0241 (11)	
H12A	1.5153	0.9944	0.6503	0.036*	
H12B	1.1887	0.9319	0.6700	0.036*	
H12C	1.5530	0.9152	0.7077	0.036*	

### Atomic displacement parameters (Å^2^)

**Table:**

	*U*^11^	*U*^22^	*U*^33^	*U*^12^	*U*^13^	*U*^23^
Br1	0.0245 (3)	0.0267 (3)	0.0331 (3)	−0.0011 (2)	0.0081 (2)	0.0102 (2)
N1	0.027 (2)	0.024 (2)	0.023 (2)	0.0019 (18)	0.0048 (17)	0.0007 (18)
N2	0.018 (2)	0.0173 (19)	0.024 (2)	0.0029 (16)	0.0018 (16)	−0.0005 (16)
N3	0.018 (2)	0.016 (2)	0.025 (2)	0.0009 (16)	0.0031 (16)	0.0015 (16)
N4	0.021 (2)	0.018 (2)	0.027 (2)	−0.0009 (17)	0.0047 (16)	0.0024 (17)
C1	0.017 (2)	0.023 (2)	0.025 (2)	0.0025 (19)	0.0039 (18)	0.008 (2)
C2	0.024 (3)	0.019 (2)	0.029 (3)	−0.001 (2)	0.005 (2)	−0.002 (2)
C3	0.019 (2)	0.022 (2)	0.023 (2)	−0.0002 (19)	0.0032 (19)	−0.001 (2)
C4	0.017 (2)	0.020 (2)	0.020 (2)	0.0028 (19)	0.0033 (17)	0.0016 (19)
C5	0.023 (2)	0.022 (2)	0.022 (2)	0.0055 (19)	−0.0005 (19)	−0.0042 (19)
C6	0.015 (2)	0.020 (2)	0.021 (2)	0.0061 (18)	0.0003 (18)	0.0002 (19)
C7	0.025 (3)	0.016 (2)	0.022 (2)	0.0009 (19)	0.0065 (19)	−0.0035 (19)
C8	0.016 (2)	0.015 (2)	0.021 (2)	0.0013 (17)	0.0026 (18)	0.0006 (18)
C9	0.017 (2)	0.019 (2)	0.026 (2)	0.0029 (19)	0.0026 (19)	0.003 (2)
C10	0.027 (3)	0.019 (2)	0.031 (3)	−0.001 (2)	0.001 (2)	0.001 (2)
C11	0.020 (2)	0.013 (2)	0.034 (3)	0.0008 (18)	0.002 (2)	−0.003 (2)
C12	0.024 (3)	0.027 (3)	0.021 (2)	0.002 (2)	0.0069 (19)	0.000 (2)

### Geometric parameters (Å, °)

**Table:**

Br1—C1	1.905 (5)	C3—H3	0.9500
N1—C1	1.316 (7)	C4—C5	1.381 (7)
N1—C5	1.347 (7)	C4—C6	1.458 (7)
N2—C8	1.340 (6)	C5—H5	0.9500
N2—C6	1.371 (6)	C6—C7	1.393 (7)
N3—C7	1.359 (6)	C7—H7	0.9500
N3—C8	1.382 (6)	C8—C9	1.424 (7)
N3—C11	1.386 (6)	C9—C12	1.482 (7)
N4—C9	1.327 (6)	C10—C11	1.335 (7)
N4—C10	1.371 (7)	C10—H10	0.9500
C1—C2	1.384 (7)	C11—H11	0.9500
C2—C3	1.369 (7)	C12—H12A	0.9800
C2—H2	0.9500	C12—H12B	0.9800
C3—C4	1.401 (7)	C12—H12C	0.9800
			
C1—N1—C5	116.8 (4)	C7—C6—C4	126.6 (4)
C8—N2—C6	104.9 (4)	N3—C7—C6	105.4 (4)
C7—N3—C8	107.6 (4)	N3—C7—H7	127.3
C7—N3—C11	132.2 (4)	C6—C7—H7	127.3
C8—N3—C11	120.2 (4)	N2—C8—N3	111.1 (4)
C9—N4—C10	118.5 (4)	N2—C8—C9	130.2 (4)
N1—C1—C2	125.0 (5)	N3—C8—C9	118.7 (4)
N1—C1—Br1	115.9 (4)	N4—C9—C8	120.4 (4)
C2—C1—Br1	119.1 (4)	N4—C9—C12	119.8 (5)
C3—C2—C1	117.3 (5)	C8—C9—C12	119.9 (4)
C3—C2—H2	121.3	C11—C10—N4	124.6 (5)
C1—C2—H2	121.3	C11—C10—H10	117.7
C2—C3—C4	120.2 (5)	N4—C10—H10	117.7
C2—C3—H3	119.9	C10—C11—N3	117.6 (5)
C4—C3—H3	119.9	C10—C11—H11	121.2
C5—C4—C3	117.0 (4)	N3—C11—H11	121.2
C5—C4—C6	121.0 (4)	C9—C12—H12A	109.5
C3—C4—C6	122.0 (4)	C9—C12—H12B	109.5
N1—C5—C4	123.8 (5)	H12A—C12—H12B	109.5
N1—C5—H5	118.1	C9—C12—H12C	109.5
C4—C5—H5	118.1	H12A—C12—H12C	109.5
N2—C6—C7	110.9 (4)	H12B—C12—H12C	109.5
N2—C6—C4	122.4 (4)		

### Hydrogen-bond geometry (Å, °)

**Table:**

*D*—H···*A*	*D*—H	H···*A*	*D*···*A*	*D*—H···*A*
C7—H7···N1^i^	0.95	2.52	3.438 (6)	163
C10—H10···N2^ii^	0.95	2.60	3.484 (7)	156

Symmetry codes: (i) −*x*+1, −*y*+2, −*z*+2; (ii) −*x*+5/2, *y*+1/2, −*z*+3/2.

## References

[bb1] Bruker (2001). *SADABS* Bruker AXS Inc., Madison, Wisconsin, USA.

[bb2] Bruker (2007). *APEX2 *and *SAINT* Bruker AXS Inc., Madison, Wisconsin, USA.

[bb3] Lumma, W. C. Jr & Springer, J. P. (1981). *J. Org. Chem.* **46**, 3735–3736.

[bb4] Sheldrick, G. M. (2008). *Acta Cryst.* A**64**, 112–122. 10.1107/S0108767307043930 18156677

